# Reversion of P-Glycoprotein-Mediated Multidrug Resistance in Human Leukemic Cell Line by Diallyl Trisulfide

**DOI:** 10.1155/2012/719805

**Published:** 2012-07-12

**Authors:** Qing Xia, Zhi-Yong Wang, Hui-Qing Li, Yu-Tao Diao, Xiao-Li Li, Jia Cui, Xue-Liang Chen, Hao Li

**Affiliations:** ^1^Department of Hematology, Qilu Hospital, Shandong University, Shandong, Jinan 250012, China; ^2^Department of Emergency, Qilu Hospital, Shandong University, Shandong, Jinan 250012, China; ^3^Institute of Basic Medicine, Shandong Academy of Medical Sciences, Shandong, Jinan 250062, China; ^4^Soochow University and Department of Hematology, Branch Guangci of First Affiliated Hospital, Jiangsu, Suzhou 215128, China; ^5^Shouguang Centre for Disease Control and Prevention, Shouguang 262700, China

## Abstract

Multidrug resistance (MDR) is the major obstacle in chemotherapy, which involves multiple signaling pathways. Diallyl trisulfide (DATS) is the main sulfuric compound in garlic. In the present study, we aimed to explore whether DATS could overcome P-glycoprotein-(P-gp-)mediated MDR in K562/A02 cells, and to investigate whether NF-**κ**B suppression is involved in DATS-induced reversal of MDR. MTT assay revealed that cotreatment with DATS increased the response of K562/A02 cells to adriamycin (the resistance reversal fold was 3.79) without toxic side effects. DATS could enhance the intracellular concentration of adriamycin by inhibiting the function and expression of P-gp, as shown by flow cytometry, RT-PCR, and western blot. In addition, DATS resulted in more K562/A02 cell apoptosis, accompanied by increased expression of caspase-3. The expression of NF-**κ**B/p65 (downregulation) was significantly linked to the drug-resistance mechanism of DATS, whereas the expression of I**κ**B**α** was not affected by DATS. Our findings demonstrated that DATS can serve as a novel, nontoxic modulator of MDR, and can reverse the MDR of K562/A02 cells in vitro by increasing intracellular adriamycin concentration and inducing apoptosis. More importantly, we proved for the first time that the suppression of NF-**κ**B possibly involves the molecular mechanism in the course of reversion by DATS.

## 1. Introduction

The multidrug resistance (MDR) of leukemic cells to chemotherapy remains the most significant cause of treatment failure in acute leukemia [1]. A number of studies have shown that the major contributor to MDR is increased drug efflux mediated by P-glycoprotein (P-gp), a product of the mdr-1 gene [2, 3]. Enormous efforts have been exerted to find reversal agents of the drug-efflux pump to overcome MDR [4]. Although hundreds of compounds have been found to reverse MDR, their clinical application is limited due to unacceptable side effects or toxicity at the doses required for effectiveness [5, 6]. Therefore, searching for reversal agents with low toxicity and high reversal activity has become an important research task.

Many plant-derived drugs or herbal formulations have been proven to have antitumor potential in vitro and in vivo [7–9]. Many of them, such as carnosic acid, puerarin, and ampelopsin, may reverse P-gp-mediated MDR via a decrease in the expression of mdr-1 in K562/A02 cells, which is an adriamycin-selective, Pgp-overexpressing subline [10–12]. However, there have been several reports which claimed that some of the plant-derived drugs could increase the P-GP function [13].

Diallyl trisulfide (DATS) is the main sulfuric compound in garlic. Garlic compounds have been shown to have antiviral, antibacterial, antioxidant, anti-inflammatory, antiproliferative, and antiangiogenic activities [14–19]. In vitro and in vivo preclinical studies have implicated DATS as an important mediator of cyclins and cell cycle arrest, apoptosis, cell adhesion, and angiogenesis [20–27]. Engdal proposed that garlic compounds could inhibit P-gp expression in vitro and in vivo [28]. DATS may have the ability to reverse drug resistance, but its molecular mechanism is still not fully understood.

We evaluated the P-gp-modulating potential of DATS in MDR K562/A02 cells to prove the effect of DATS as a reversal agent for human leukemic cells in vitro. In our previous study, the IC10 of DATS to K562/A02 cells was 2 *μ*mol/L, a noncytotoxic concentration dose also used in the present study. A verapamil-treated (4 *μ*g/mL) group was used as positive control. Our goal was to ascertain whether transcription nuclear factor B (NF-*κ*B) activation is involved in the reversal mechanism of DATS.

## 2. Materials and Methods

### 2.1. Drugs and Reagents

The following compounds were purchased: DATS (Shandong Lukang Xin Chen Pharmaceutical Co., Ltd., Shandong, China), verapamil (Shanghai Harvest Pharmaceutical Co., Ltd., Shanghai, China), adriamycin (ADM, Sigma Chemical Co., MO, USA), annexin V-FITC/PI (JingMei Bioengineering Co., Ltd., China), mouse antihuman P-gp monoclonal (BD Pharmingen Co., Ltd., USA), mouse antihuman mdr-1 antibody (Chemicon Co., Ltd., USA), rabbit anti-NF-*κ*B (P65) antibody and mouse anti-human I*κ*B*α* antibody (Cell Signaling Co., Ltd., USA), and caspase-3 rabbit antihuman monoclonal antibody (Beijing Biosynthesis Biotechnology Co., Ltd., Beijing, China). The primers were synthesized by Shanghai Boshang Biotechnology Co., Ltd. (Shanghai, China).

### 2.2. Cell Lines and Cell Culture

Human leukemia cell line K562 and its adriamycin-selective, Pgp-overexpressing subline K562/A02 were obtained from the Institute of Basic Medicine, Shandong Academy of Medical Sciences, China. Both cell lines were cultured in RPMI1640 medium (Gibco, Los Angeles, CA, USA) supplemented with 10% (v/v) heat-inactivated newborn calf serum (HangZhou Sijiqing Biological Engineering Materials Co., Ltd., China), 100 U/mL penicillin, and 100 *μ*g/mL streptomycin. Furthermore, both cell lines were grown in a humidified incubator at 37°C and 5%CO_2_. In particular, the K562/A02 cell line was maintained in 1 *μ*g/mL adriamycin-containing medium and incubated in adriamycin-free medium for 2 weeks before the experiments.

### 2.3. Assay of In Vitro Drug Sensitivity

3-(4,5-Dimethylthiazol-2-yl)-2,5-diphenyltetrazoliumbromide (MTT) assay was used to compare the MDR of the K562 and K562/A02 cells to adriamycin. K562 and K562/A02 cells were grown in a 96-well plate at 1 × 10^5^ cells/mL in a complete RPMI 1640 medium. Adriamycin was then added at various drug concentrations. K562 and K562/A02 cells without drugs in the medium were used as the blank controls. After the treatments were incubated for 44 h at 37°C, 20 *μ*L MTT was added into each well, and incubation continued for another 4 h. The medium was then removed, and 150 *μ*L dimethyl sulfoxide was added to each well to dissolve the formazan crystals. The absorbance value was measured with a spectrophotometer at the wavelength of 570 nm. IC50 of the drugs was calculated based on the MTT assay

(1) Inhibition  of  cell  viability =1−average  A  value  of  experimental  groupaverage  A  value  of  blank  control  group×100%,Drug  resistance  fold=IC50  of  drug-resistant  groupIC50  of  sensitive  group.

K562/A02 cells were seeded into 96-well culture plates at a density of 1 × 10^5^ cells/mL to determine whether DATS can sensitize MDR cells to the cytotoxicity of adriamycin. Adriamycin was then added with varying concentrations. The experimental group was treated with DATS at a concentration of 2 *μ*mol/L. Verapamil (4 mg/L) treatment served as positive control. The cells were analyzed using the MTT method

(2) $$\begin{array}{c} \text{Reverse}\;\;\text{fold}=\frac{\text{IC}50\;\;\text{before}\;\;\text{reversal}}{\text{IC}50\;\;\text{after}\;\;\text{reversal}}.\; \end{array}$$

### 2.4. Detection of Intracellular Adriamycin Concentration by FCM

The adriamycin concentration in K562/A02 cells was measured by flow cytometry (FCM). The K562 cells and K562/A02 cells without treatment were used as negative control. K562/A02 cells were treated with 2 *μ*mol/L DATS or 4 mg/L verapamil and incubated for 48 h [29]. Adriamycin was then added to each sample to a final concentration of 5 mg/L. After incubating further for 2 h [30], the cells were harvested by centrifugation, washed twice with ice-cold phosphate buffered solution (PBS), and then resuspended in PBS. Adriamycin was excited effectively at a single wavelength (488 nm), and the emitted light was collected in the fluorescence-3 (FL3) channel. Events were gated on an FSC versus SCC dot plot to exclude the influences of cell debris and aggregates. A total of 10000 gated cells were detected for each sample, which were analyzed by Modfit LT software.

### 2.5. Detection of P-gp Expression by FCM

P-gp expression on the surface membrane of K562/A02 cells was determined by a direct immunofluorescence staining technique. K562/A02 cells were cocultured with 2 *μ*mol/L DATS or 4 mg/L verapamil for 48 h. They were washed twice and suspended in PBS. The cell suspension was incubated with phycoerythrin-conjugated UIC2, mouse antihuman P-gp monoclonal antibody (P-gp-PE), and the homotype control IgG2a-PE. The mixture was reacted at room temperature and away from light for 30 min, washed twice, and then detected by FCM. The specific P-gp antibody UIC2 was detected in the FL-2 channel; thus, P-gp expression can be assessed on the cell surface. Protein expression was analyzed using Cell Quest software.

### 2.6. Observation of Morphological Changes by Light Microscopy

The morphological changes of apoptotic cells with hematoxylin-eosin (HE) staining were observed by light microscopy. K562/A02 cells were grown at 1 × 10^5^ cells/mL in a complete RPMI 1640 medium on a 24-well plate with a coverslip set at the bottom. After treatment with 2 *μ*mol/L DATS combined with a final concentration of 1 mg/L adriamycin for 24 h to 72 h, (treatment with adriamycin alone as control), the coverslip was removed in each experimental group. Using 95% ethanol, the coverslip contents were fixed for 20 min. A series of washings were then performed as follows: carefully washed with PBS two times, hematoxylin for 2 min to 3 min, water to wash away hematoxylin for 1 s to 3 s, 1% hydrochloric acid and ethanol for 2 s to 3 s to reduce the cytoplasmic stain, lightly washed for 10 s, ammonia for 10 s to 20 s, and running water for 10 s. Eosin staining was performed for 1 m, then the stain was removed by washing with water for 1 s to 2 s, 80% ethanol 1 s to 2 s, 95% ethanol for 3 min to 5 min, ethanol for 5 min to 10 min, xylene (I) for 3 min to 5 min, and xylene (II) for 2 min to 5 min. The coverslip was then air-dried and mounted with neutral gum. Finally, the specimen was observed by light microscopy.

### 2.7. Apoptosis Assay by Statistical FCM

The apoptosis rates were measured using flow cytometric assay. Cell labeling was performed using annexin V conjugated to FITC, which binds to phosphatidylserine exposed on the surface membrane of cells undergoing apoptosis. After incubation in the medium containing different drugs (2 *μ*mol/L DATS or 4 mg/L verapamil) at 37°C for 48 h, the cell suspensions were washed twice with PBS and centrifuged at 550× g for 5 min. The cells were suspended in 500 *μ*L binding buffer, 5 *μ*L annexin V-FITC, and 10 *μ*L (20 *μ*g/mL) PI solution incubated at room temperature for 15 min in the dark. The samples were measured using a flow cytometer with FACS software.

### 2.8. Semiquantitative RT-PCR Assay

After treatment with the drugs (2 *μ*mol/L DATS or 4 mg/L verapamil), in vitro total mRNA was extracted from the cells with trizol reagent (Invitrogen Co., CA, USA) according to the manufacturer's instructions. Single-stranded cDNA was synthesized by reverse transcription from 1 *μ*g of the total RNA using reverse transcriptase RNAse M-MLV (Invitrogen Co., CA, USA) and oligo-dT. The amplification was performed in a final volume of 50 *μ*L, containing 5 *μ*L cDNA, 0.5 *μ*L of each oligonucleotide primer, 1 *μ*L of each dNTP, and 1 unit of Taq DNA polymerase. Amplification was carried out in a thermal cycler. The PCR primers and expected product size were as follows: mdr-1 (167bp) were 5′-0-3′ (forward) and 5′-GTTCAAACTTCTGCTCCTCA-3′ (reverse); *β*-actin (540bp) were -GTGGGGCGCCCCAGGCACCA-3′ (forward) and 5′-CTCCTTAATGTCACGCACGATTTC-3′ (reverse); NF-*κ*B/p65 (293bp) were 5′-TGCACCTAGCTGCCAAAGAAGGA-3′ (forward) and 5′-TCTGCTCCTGCTGCTTTGAGAA-3′ (reverse); I*κ*B*α* (634bp) were 5′-GCAGAGGATTACGAGCAGAT-3′ (forward) and 5′-CCTGGTAGGTTACTCTGTTG-3′ (reverse); Caspase-3 (358 bp) were 5′-CCCATTTCTCCATACGCACT-3′ (forward) and 5′-TGACAGCCAGTGAGACTTGG-3′ (reverse). The circulating conditions were as follows: 94°C for 1 min, 58°C for 1 min, and 72°C for 1 min for 26 cycles, and then extended for 7 min at 72°C. The products were identified by electrophoresis using 1.5% agarose gel. Using *β*-actin as internal reference, the products were further analyzed using Alpha gel image analysis system.

### 2.9. Western Blot Analysis of Protein Expression

After treatment with the drugs (2 *μ*mol/L DATS or 4 mg/L verapamil), total protein was isolated and subjected to sodium dodecyl sulfate PAGE analysis and transferred to a polyvinylidene difluoride membrane. The blots were stained with primary antibodies (1 : 1000–1200, mouse anti-human mdr-1 antibody, rabbit anti-human NF-*κ*B (P65) antibody, mouse anti-human I*κ*B*α* antibody, and rabbit anti-human caspase-3 monoclonal antibody) overnight at 4°C, and then with horseradish peroxidase-conjugated goat antirabbit IgG (1 : 5000) for 1 h at room temperature. The signal was detected with an ECL Western blot detection kit (ZhongShan Co., Beijing, China). After normalization by the corresponding *β*-actin expression, protein expression level was determined by densitometry scans and measured with Quantity One software.

### 2.10. Analyses

Statistical calculations were carried out with SPSS 17.0 for Windows software package. The results were expressed as mean ± standard deviation of three independent experiments. Student's *t*-test was used for the statistical analyses, and *P* values < 0.05 were considered significant. The synergetic effect of the two drugs was analyzed using factorial analysis.

## 3. Results

### 3.1. Drug Sensitivity

**Figure 1:**
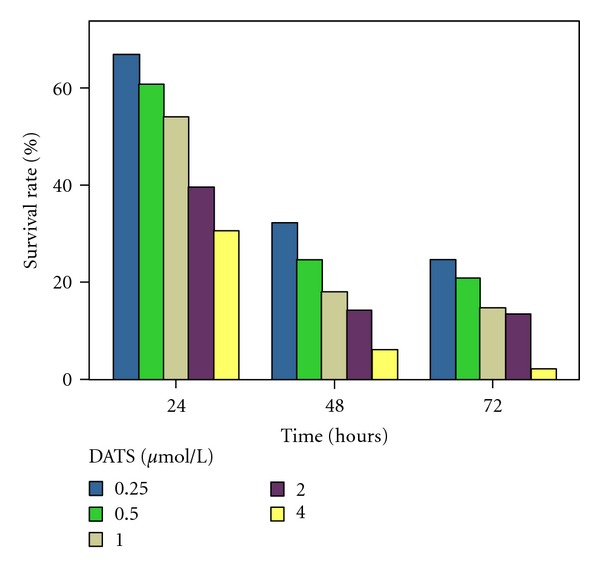
The time- and concentration-dependent reversal effects of DATS on the K562/A02 cells. K562/A02 cells were treated with adriamycin (5 ug/mL) for varying time intervals (24 h, 48 h, and 72 h) in the presence of 0.25– 4 *μ*mol/L DATS.

MTT assay was used to study the cytotoxicity of adriamycin. The ability of DATS at 2 *μ*mol/L to enhance the cytotoxicity of adriamycin in K562/A02 was examined. The MTT assay results are summarized in Table 1. The IC50 value of adriamycin for K562/A02 decreased after treatment with DATS (*P* < 0.01). The time- and concentration-dependent reversal effects of DATS on the K562/A02 cells were observed for 24, 48, and 72 h. The data of the three experiments are shown in Figure 1. The higher the concentration of DATS used, the better the inhibitive effect.

### 3.2. Detection of Intracellular Adriamycin Concentration

**Figure 2:**
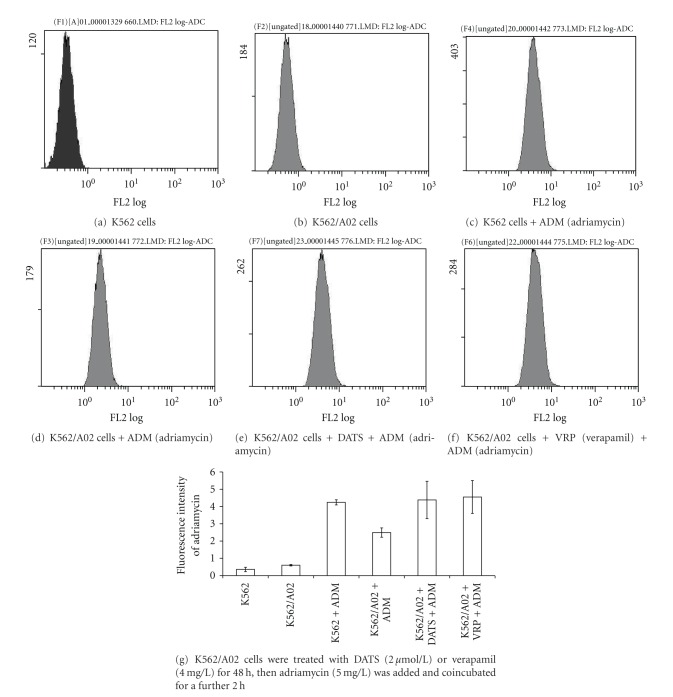
Concentration of adriamycin in K562 or K562/A02 cells.

The effect of DATS on the intracellular accumulation of adriamycin was examined by FCM. The autofluorescence intensity of the K562 and K562/A02 cells was very low (Figures 2(a) and 2(b)). The fluorescence intensity of adriamycin in K562 cells was 4.24 ± 0.15, whereas it was 2.49 ± 0.27 in K562/A02 cells (Figures 2(c) and 2(d), *P* < 0.01). After treatment with DATS, adriamycin fluorescence intensity in K562/A02 cells increased to 4.38 ± 1.08 (Figure 2(e)), showing a significant difference compared with that without DATS treatment cells (*P* < 0.01). These results showed that DATS can enhance the intracellular concentration of adriamycin.

### 3.3. Alteration of P-gp Expression

**Figure 3:**
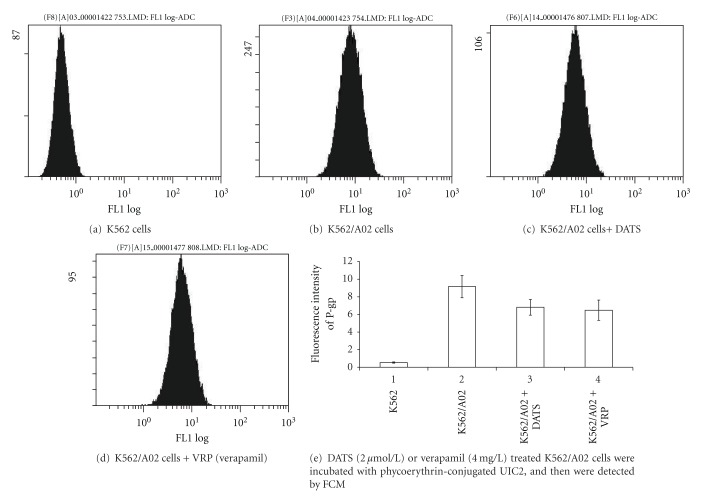
The expression of P-gp on the membrane surface of K562 or K562/A02 cells.

DATS-treated K562/A02 cells were incubated with phycoerythrin-conjugated UIC2, and then detected by FCM. The expression of P-gp in K562 was lower than that in K562/A02 (*P* < 0.01). After treatment with DATS in K562/A02 cells, P-gp expression decreased. A significant difference was observed between untreated K562/A02 cells and treated K562/A02 cells (*P* < 0.01) (Figure 3).

### 3.4. Apoptosis Observed by Light Microscopy

**Figure 4:**
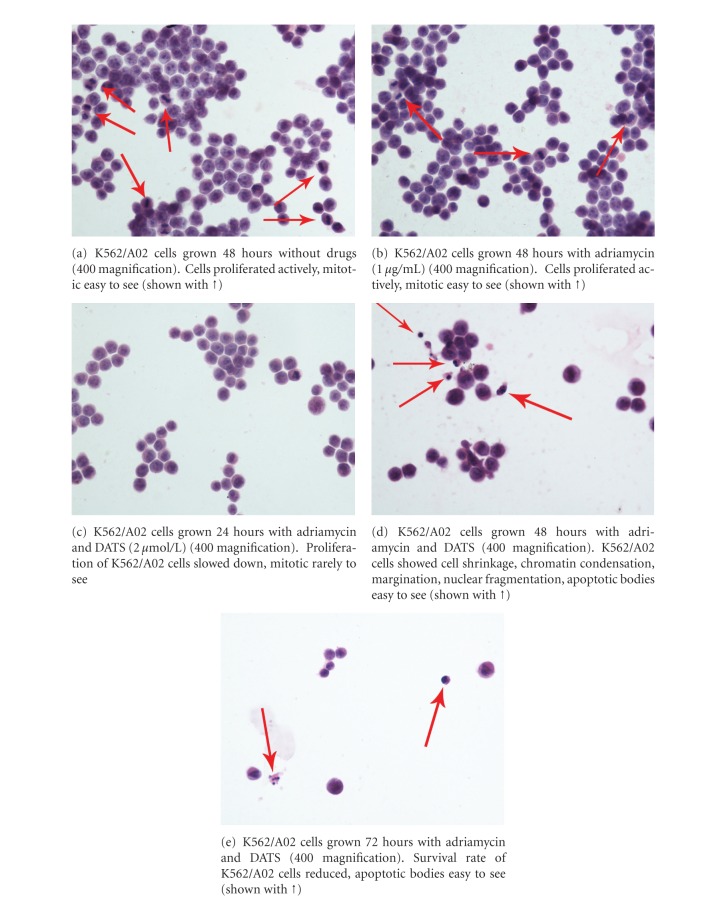
Morphology observed by light microscopy.

Adriamycin (1 *μ*g/mL) alone did not inhibit the proliferation of K562/A02 cells significantly (Figures 4(a) and 4(b)). After simultaneous treatment of adriamycin (1  *μ*g/mL) with DATS (2 *μ*mol/L) for 24 h, the proliferation of K562/A02 cells slowed down (Figure 4(c)). After 48 h, the K562/A02 cells showed cell shrinkage, chromatin condensation, margination, nuclear fragmentation, apoptotic bodies, and typical apoptotic cytomorphological features (Figure 4(d)). After 72 h, more apoptotic cells and less surviving K562/A02 cells were detected (Figure 4(e)).

### 3.5. Apoptosis Statistical FCM Assay

**Figure 5:**
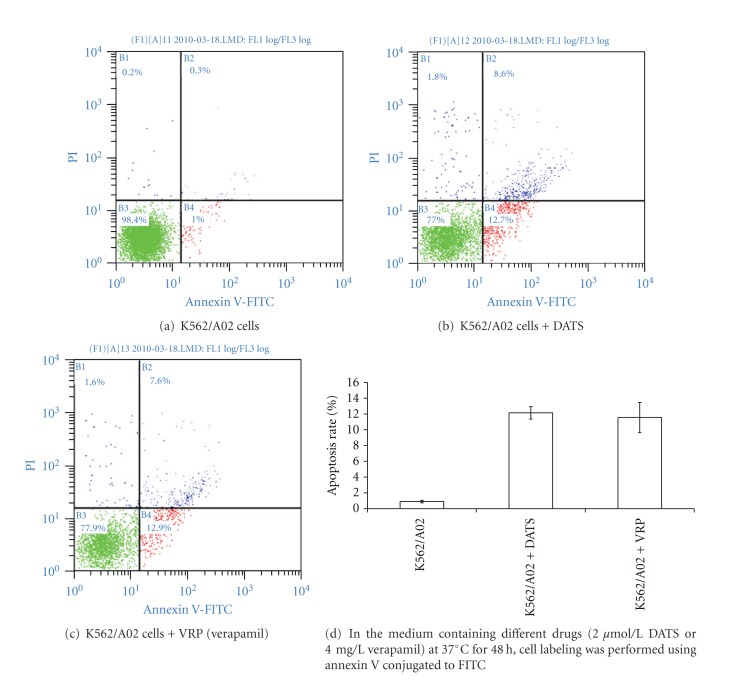
Apoptosis of K562/A02 cells induced by DATS or verapamil.

Apoptosis of K562/A02 cells was induced by DATS or verapamil. After incubation with either DATS (2 *μ*mol/L) or verapamil (4 *μ*g/mL) for 48 h, apoptotic percentages of K562/A02 cells were 12.15 ± 0.78% and 11.55 ± 1.91%, respectively. Evident differences were found compared with the control (0.9 ± 0.17%, *P* = 0.000) (Figure 5).

### 3.6. Detection of Gene Expression

**Figure 6:**
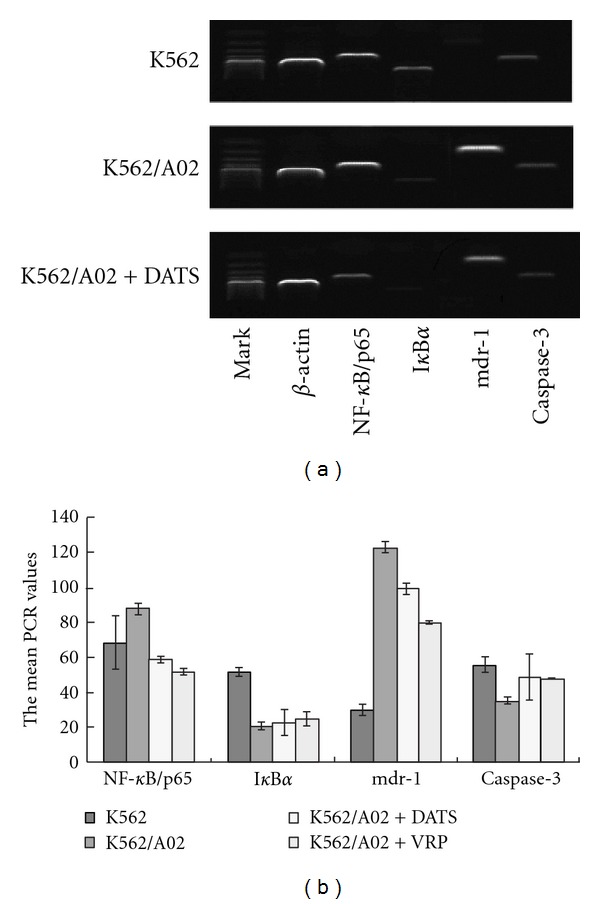
The mean PCR values of the ratio relative to the *β*-actin gene DATS (2 *μ*mol/L) or verapamil (4 mg/L) treated K562 cells and K562/A02 cells.

As demonstrated by semiquantitative RT-PCR, overexpression of mdr1 mRNA was detected better in the K562/A02 cells compared with the K562 cells (*P* < 0.05). The K562/A02 cells expressed high-levels of NF-*κ*B/p65, but expressed low-levels of I*κ*B*α* and caspase-3. DATS could downregulate the expression of mdr1 and NF-*κ*B/p65 (*P* < 0.05) and upregulate the expression of caspase-3 (*P* < 0.05). However, DATS cannot evidently increase the expression level of I*κ*B*α* (*P* > 0.05) (Figure 6).

### 3.7. Western Blot Analysis of Protein Expression

**Figure 7:**
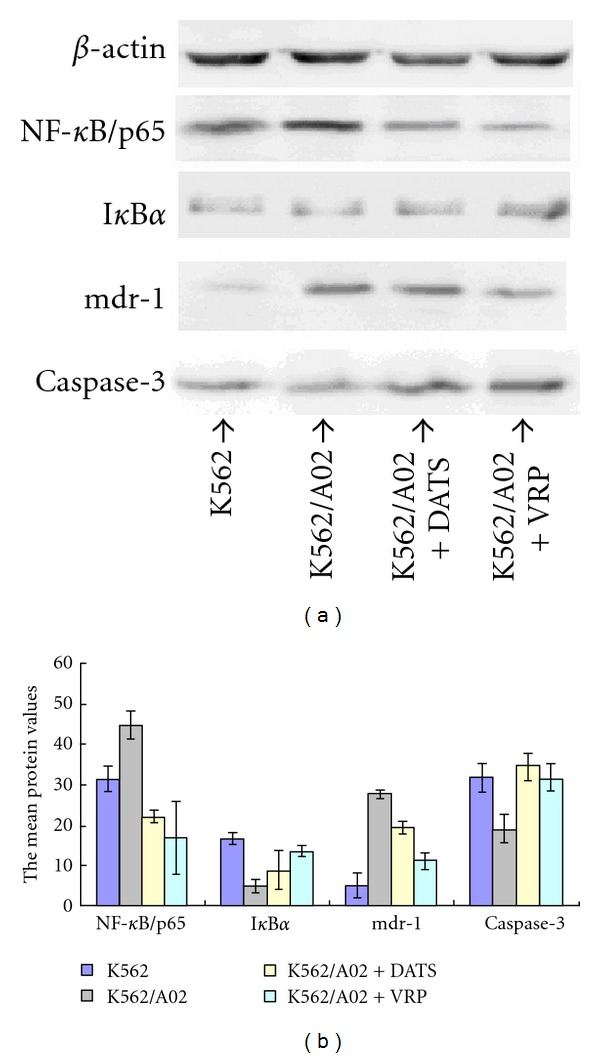
The mean protein values of the ratio relative to the *β*-actin DATS (2 *μ*mol/L) or verapamil (4 mg/L) treated K562 cells and K562/A02 cells.

The Western blot report revealed that the expression of mdr1 protein and NF-*κ*B/p65 protein were much higher in K562/A02 cells compared with those in K562 cells (*P* < 0.05). The expression level of I*κ*B*α* protein and caspase-3 protein in K562/A02 cells was much lower than that in K562 cells (*P* < 0.05). DATS could downregulate the expression of mdr1 and NF-*κ*B/p65 (*P* < 0.05) and upregulate the expression of caspase-3 (*P* < 0.05). However, DATS cannot evidently increase the expression level of I*κ*B*α* (*P* > 0.05) (Figure 7).

## 4. Discussion

In the present study, we have shown that DATS can significantly reverse the MDR of K562/A02 cells in vitro by increasing intracellular adriamycin concentration through downregulating the overexpression of P-gp and inducing apoptosis via increased caspase-3 expression. More importantly, we have proved that the NF-*κ*B signaling pathway is involved in the reversal mechanism of DATS.

The active efflux of xenobiotics is a major mechanism of cell adaptation to environmental stress. The ATP-dependent transmembrane transporter Pg-p confers long-term cell survival in the presence of different toxins, including anticancer drugs.

P-gp, a product of the mdr-1 gene, is a 170 kDa ATP-dependent transmembrane transporter that acts as a drug efflux pump, decreasing intracellular drug accumulation and therefore reducing intracellular drug efficacy [2, 3]. A high P-gp expression level is usually observed in MDR cell lines. However, in sensitive cells, P-gp is usually not detected. The drug intake capacity of cells with high mdr-1 gene expression levels have no difference compared with sensitive cells, but drug efflux capacity is significantly increased [31, 32].

MDR is one of the major causes of failure in leukemic chemotherapy and is associated with the overexpression of P-gp in leukemic cell membranes. Studies have shown that in acute myeloid leukemia patients, the clinical remission (CR) rate of original leukemia cells with P-gp expression is 50%, whereas the CR rate of P-gp-negative cells is 81%. The P-gp expression in recrudescent leukemia group is significantly higher than in the first treatment group.

Enormous efforts have been exerted to find reversal agents of the drug efflux pump and overcome MDR. Various compounds, such as verapamil, cyclosporin, quinidine, tamoxifen, progesterone, reserpine, and others, have been reported to overcome MDR in vitro by decreasing mdr-1 expression [4]. However, their clinical application is limited due to their unacceptable side effects or toxicity at the doses required for effectiveness.

Plant-derived drugs or herbal formulations have multitarget functions and have low toxicity. Recently, traditional Chinese medicine and its extracts have been shown to have high reversal activity on MDR. Yu et al. proved that carnosic acid (CA) can reverse the MDR of K562/A02 cells in vitro by increasing intracellular adriamycin concentration, downregulating the expression of mdrl, and inhibiting the function of P-gp [10]. Chen et al. found that puerarin can reverse the multidrug resistance of K562/A02 to ADR by inhibiting the expression of p-gp and survivin [11]. Ye discovered that ampelopsin (AMP) could increase the cytotoxicity and intracellular accumulation of chemotherapeutic drugs in MDR-associated tumor cells by inhibiting the efflux of drugs by P-gp [12].

DATS is the main sulfuric compound of garlic, and garlic has been confirmed to be beneficial to human health via multiple mechanisms. Arora et al. discovered that nontoxic concentration of DATS enhances the cytotoxic effects of VBL and VCR in VBL-resistant human leukemia (K562/R) cells in a time-dependent manner by decreasing the expression levels of P-gp [33]. However, Lai et al. proved that DAS, DADS, and DATS promote expression of mdr1 genes in colo 205 human colon cancer cells [34]. Therefore, the reversal effect of DATS to multidrug resistance is still controversial. 

 In the current study, we showed through MTT assays that DATS enhances the toxicity of adriamycin in K562/A02. The MDR leukemia cell line K562/A02 was established through in vitro selection of K562 cells with an increasing concentration of adriamycin [35] overexpressing the mdr1 gene and P-gp. DATS could decrease the IC50 of adriamycin against K562/A02 cells, as well as increase its chemosensitivity, which means DATS could reverse MDR. The fluorescence intensity value of adriamycin measured via FCM reinforced the finding that DATS could increase the toxicity of adriamycin by increasing intracellular adriamycin concentration.

We used FCM to detect P-gp protein expression on the K562/A02 cell surface, which determined whether DATS could reduce P-gp expression. After treatment with DATS, P-gp expression decreased significantly, which demonstrated that DATS could reverse MDR by inhibiting P-gp overexpression. The decreased expression of P-gp was consistent with the downregulation of the mdr1 gene and protein, as demonstrated by RT-PCR and Western blot. The result was similar to the report of Arora. But its molecular mechanism is still not fully understood.

We found that the intracellular adriamycin concentration in DATS-treated K562/A02 cells was 1.76-fold that in untreated K562/A02 cells. The extent of drug resistance reversal to adriamycin of DATS-treated K562/A02 cells was 3.79-fold that of untreated K562/A02 cells. Thus, other factors probably play important roles in the MDR mechanism, besides the reduction of intracellular drug accumulation. 

In recent years, studies have shown that apoptosis is closely related to MDR. Many anticancer drugs with different structures and different targets can induce apoptosis in tumor cells. The mechanism of apoptosis may be involved in the mechanism of MDS. Apoptosis is the common pathway of many different drugs. Due to the inhibition of apoptosis, the tumor cells are not only resistant to a certain drug, but are resistant to other drugs at the same time. This phenomenon leads to MDR.

We compared the morphological changes of cells with HE staining by light microscopy. As shown in Figure 4, the effect of DATS combined with a small dose of chemotherapeutic agent on K562/A02 cells resulted in strikingly increased apoptosis. At the same time, the apoptosis rate of K562/A02 cells induced by DATS was measured using flow cytometric assay. The proapoptotic mechanism played an important role in the process of reversal of MDR by DATS, as revealed by evidence.

The caspase family, a large class of proteases, plays a very important role in the activation and execution of apoptosis. Thus, some researchers have named them the effectors of apoptosis. In the caspase family, caspase-3 is the key element of implementation because it participates in many apoptotic signaling pathways. The weakened expression of caspase-3 has been associated with drug resistance and apoptotic inhibition in hematologic malignancies [36, 37]. The induced cell death in K562-Vinc cells was associated with activation of caspase-3 [38]. Several studies have documented that garlic can increase the activation of caspase-3 [39–41]. The current study arrived at the same conclusion. The ratio of caspase-3 expression in drug-resistant strains was significantly lower than in the sensitive strains. DATS could upregulate caspase-3 protein significantly in K562/A02 cells, which means DATS can promote apoptosis by adjusting caspase-3 expression.

As revealed in the current experiment, DATS can reverse the MDR of K562/A02 cells by increasing intracellular adriamycin concentration via downregulating the overexpression of P-gp and inducing apoptosis by activating increased caspase-3 expression. However, no research has yet indicated the signaling pathway through which DATS can adjust these genes.

 The importance of NF-*κ*B in the process of cancerization has been mentioned in previous research. NF-*κ*B proteins are a small group of related and evolutionarily conserved proteins, in which mammals consists of five members, namely, Rel (c-Rel), RelA/p65, RelB, p50, and p52. In resting cells, NF-*κ*B is sequestered in the cytoplasm through the inhibitory molecule, termed inhibitor of NF-*κ*B (I*κ*B), such as I*κ*B*α*, which masks the nuclear localization sequence of NF-*κ*B. The stimulation of cells with various stimuli, including the stimulation of the TCR signaling pathway, leads to the activation of the I*κ*B kinase (IKK). NF-*κ*B signaling can be dichotomized into a “classical” pathway in which I*κ*B kinase (IKK*β*) phosphorylates I*κ*B*α*. An “alternative” NF-*κ*B pathway exists, in which IKK*α* phosphorylates the p100 precursor of the NF-*κ*B p52 subunit. The activated IKK phosphorylates I*κ*B, triggering rapid ubiquitination and degradation of I*κ*B by the 26S proteasome complex, which unmasks the nuclear localization sequence of NF-*κ*B. Therefore, NF-*κ*B can be rapidly translocated into the nucleus to initiate the transcription of its target genes. The result of these signaling events is the accumulation of the heterodimeric NF-*κ*B transcription factors in the nucleus, with the classical pathway regulating mainly p50/p65 and p50/c-Rel dimers, and the alternative pathway regulating p52/relB dimers [42].

NF-*κ*B can intervene in oncogenesis by regulating the expression of a large number of genes that regulate apoptosis, cell proliferation and differentiation, as well as inflammation, angiogenesis, and tumor migration [43]. Therefore, the inhibition of NF-*κ*B has been proposed as an adjuvant therapy for cancer. Many Phase I and II clinical studies involving different inhibitors are underway. Previous studies have proven that some Chinese medicines, such as Guan-Jen-Huang, can induced apoptosis by inhibiting NF-*κ*B activation [44].

A constitutive NF-*κ*B activity has been observed in several hematological malignancies, and this is associated with its antiapoptotic role [45, 46]. NF-*κ*B could participate in the chemoresistance of tumor cells mediated by the expression of the MDR protein. As previously confirmed, NF-*κ*B can increase the MDR gene expression in tumor cells. A purified NF-*κ*B binding sequence (5′2 CCTTTCGGGG23′) was found in the first exon of the mdr-1 promoter region, which confirmed that there are binding sites of NF-*κ*B in the mdr-1 gene. Mdr-1 may thus be NF-*κ*B downstream genes [47]. Furthermore, previous literature has confirmed that anticancer drugs, such as adriamycin and other chemicals, can damage tumor cell DNA, which can lead to the activation of NF-*κ*B. Activated NF-*κ*B promotes the transcription of mdr-1 via NF-*κ*B binding sites. Therefore, if the expression of NF-*κ*B can be inhibited, the sensitivity of chemotherapy can be increased [11, 48–50].

We have proved that K562/A02 cells display higher levels of NF-*κ*B/p65 protein expression than K562 cells. DATS can regulate the expression of NF-*κ*B/p65. In K562/A02 cells, the expression of P-gp and mdr-1 are positively correlated with NF-*κ*B/p65. Therefore, one of the mechanisms of NF-*κ*B antiapoptotic regulation in K562/A02 cells is correlated with mdr-1 and P-gp expression. Inhibition of NF-*κ*B activation may be involved in the reversal of MDR in K562/A02 cells by DATS.

Although the molecular mechanism of NF-*κ*B activation in leukemic stem cells or AML blasts remains elusive at present, NF-*κ*B and its unique role in the apoptotic and proliferation pathways and in drug resistance could represent an attractive target of selective drugs. NF-*κ*B inhibition has been proposed as an adjuvant therapy for cancer [47].

In conclusion, the present study has demonstrated that DATS can serve as a novel, nontoxic modulator of MDR and can reverse the MDR of K562/A02 cells in vitro by increasing intracellular adriamycin concentration, downregulating mdr-1 expression, and inducing apoptosis by activating increased caspase-3 expression. We therefore conclude for the first time that DATS can block NF-*κ*B activation, which produces the downstream inhibitory effects on chemotherapy sensitivity and apoptosis of K562/A02 cells. DATS could be a highly feasible candidate for the development of a new MDR reversal agent.

## Figures and Tables

**Table 1:** IC50 of adriamycin in K562 and K562/A02 cells.

Cell line	IC50 (ADM *μ*g/mL)	Drug resistance fold	Reverse fold
K562	0.11 ± 0.037		
K562/A02	6.79 ± 0.58	61.73^▲▲^	
K562/A02 + DATS	1.80 ± 0.348		3.79^∗∗^
K562/A02 + verapamil	0.56 ± 0.045		12.31^∗∗^

^▲▲^ *P* < 0.01 versus K562, **P < 0.01 versus K562/A02.
